# Supplementary material for: Path of career planning and employment strategy based on deep learning in the information age
Source: PLoS One. 2024 Oct 15;19(10):e0308654. doi: 10.1371/journal.pone.0308654 (PMC11478877; doi:10.1371/journal.pone.0308654)
Supplement: S1 Checklist — (PDF) [file pone.0308654.s001.pdf]

Human Participants Research Checklist

Complete the following if your study involved human participants or human participants' data. These questions should be addressed for prospective and retrospective studies.

1. Did you obtain ethics approval for this study?
   - If yes, please upload (file type "Other") the original approval document you received from your ethics committee. <u>If the original document is in another language, please also provide an English translation.</u>

     ___ Uploaded ✓ N/A

   - If you did not obtain ethical approval, please explain why this was not required below.

Ethical approval for this study and written informed consent from the participants of the study were not required in accordance with local legislation and national guidelines. This study does not involve human or animal experiments.

2. If you prospectively recruited human participants for the study – for example, you conducted a clinical trial, distributed questionnaires, or obtained tissues, data or samples for the purposes of this study, please report in the Methods:
   i. the day, month and year of the **start and end** of the recruitment period for this study.
   ii. whether participants provided informed consent, and if so, what type was obtained (for instance, written or verbal, and if verbal, how it was documented and witnessed). If your study included minors, state whether you obtained consent from parents or guardians. If the need for consent was waived by the ethics committee, please include this information.

   ___ Completed ✓ N/A

3. If you are reporting a retrospective study of medical records or archived samples, please report in the Methods section:
   i. the day, month and year when the data were accessed for research purposes
   ii. whether authors had access to information that could identify individual participants during or after data collection

   ___ Completed ✓ N/A
