# Supplementary material for: Path of career planning and employment strategy based on deep learning in the information age
Source: PLoS One. 2024 Oct 15;19(10):e0308654. doi: 10.1371/journal.pone.0308654 (PMC11478877; doi:10.1371/journal.pone.0308654)
Supplement: Attachment — Submitted filename: Response to Reviewers.docx [file pone.0308654.s002.docx]

5. Review Comments to the Author

Please use the space provided to explain your answers to the questions above. You may also include additional comments for the author, including concerns about dual publication, research ethics, or publication ethics. (Please upload your review as an attachment if it exceeds 20,000 characters)

Reviewer #1: The study idea is novel and holds potential for publication; however, there are several points that need consideration before acceptance. Firstly, the author should adhere to a standard format for the study, incorporating rationale, research questions based on identified gaps, and the study structure in the introduction section.

Answer:Based on your suggestions, the basic principles, research questions based on the discovered gaps, and research structure have been added to the introduction to ensure the completeness of the article.

Secondly, in the literature review, the author should formulate hypotheses based on the identified gaps.

Answer:Based on your opinions, after reviewing the literature, hypotheses are proposed and explained based on the gaps found, giving readers something to think about.

Thirdly, the methodology section appears significantly weak. The researcher has chosen an “experimental design” without proper justification or literature support. It is crucial for the researcher to explain the chosen experimental design, providing a detailed process and supporting literature. This should include information on how the experiments were conducted, control variables, and the overall methodology.

Answer:Based on your suggestions, an explanation of selected experimental designs has been added to the Experimental section, providing detailed procedures, including information on how the experiments were performed, control variables, and overall methodology, for easy reference by readers.

Lastly, the article lacks clarity on how the data were analyzed and what statistical tests were employed before reaching a conclusion. In summary, substantial improvements are needed for the article to meet the standards for acceptance.

Answer:Thank you for your comments. Now I would like to provide a detailed explanation of how the data was analyzed in the article and what statistical tests were used before drawing conclusions.

Reviewer #2: In the first paragraph of introduction well explained but there is a lack of literature support and evidence.

Answer:Based on your suggestions, I added literature support to the first paragraph of the introduction to improve the persuasiveness of the article.
Include recent literature evidence.

Answer:Based on your suggestion, citations for 2023 have been added to the literature.
Well explained the concepts career planning, deep learning and employment strategy but in this study there is a need to explore the relationship between these variables.

Answer:Based on your suggestions, Chapter 2 has been added to explore the relationship between variables such as career planning, deep learning and employment strategies to make it easier for readers to understand.
Need to explain what is an employment strategy and its significance in career planning.

Answer:Based on your opinions, the introduction of employment strategies and their significance in career planning has been supplemented in Section 2.2.
Explain clearly how deep learning helps to plan career with literature evidence and include more empirical evidence.

Answer：Based on your opinion, clearly explain how deep learning can help in career planning through literature evidence and including more empirical evidence in Section 2.4.1.
Where is an objective of the study?

Answer:Thank you for your opinion. The purpose of the research is introduced in the last paragraph of Chapter 1.

Provide clarity on methodology applied in the study i.e which method used or applied to analyse the data and how you choose respondents which method selected to select respondents.

Answer:Based on your suggestion, the methods applied in the study are clearly stated in Section 3.1, i.e. which method is used or applied to analyze the data, and an introduction on how to select the respondents and which method to select the respondents.
Mention the role of universities to mould career and employment strategies which boosts the study.

Answer:Thanks for your suggestions, the role of universities in shaping careers and employment strategies that promote research is detailed in Chapter 2 for the convenience of the reader.
Missing clarity on relation between career planning and deep learning. Provide clarity on it. Explain how deep learning techniques helps students to plan their career.

Answer:Provide a full explanation of the relationship between career planning and deep learning based on your recommendations. And introduce how deep learning technology can help students plan their careers.
How this study correlates job hopping with deep learning as job hopping relates individual psychological state of taking decision on job where as deep learning is a software tool.

Answer:Thank you for your suggestion. In Section 2.1, we will explain how job hopping is related to deep learning.

Career planning is a process of planning one’s career. How deep learning will act as a suitable tool to create employment to students according to their preferences.

Answer:Based on your suggestion, section 2.4.2 has been added with an introduction to how deep learning will serve as a suitable tool to create employment opportunities for students based on their preferences.
Already there exist a number of employment online platforms integrating employees and employers. What is the strategy that distinct other online platforms from that of the proposed website in the study.

Answer:Based on your suggestions, in Chapter 2, the strategies to distinguish some existing online employment platforms that integrate employees and employers from the website proposed in the study are introduced in detail to facilitate readers' comparative analysis.

Reviewer #3: I appreciate the opportunity to review this manuscript, which addresses a topic pertinent to modern educational research. However, there are several areas where the manuscript does not meet the scholarly standards expected of academic papers.

1Introduction
The current introduction lacks essential content. While it outlines the background of deep learning, it fails to provide a clear definition, identify the research gap, or articulate the novelty of the study. The introduction should also offer an explanation of the research methodology and data sources used. Given the focus on university students’ career planning and employment strategy, a rationale for selecting this specific group for study should be included.
Answer:Thank you for your comments in providing a clear definition of deep learning in the introduction and clarifying the novelty of the research.
I suggest that the authors begin the introduction with a definition of deep learning.
Answer:Based on your suggestions, I will introduce the definition of deep learning to facilitate readers' understanding of technical methods.

Literature Support
The article lacks sufficient literature support. For example, the following statement is missing corresponding references:

"Tavabie J A studied career planning for the non-clinical workforce, that is, opportunities to develop a sustainable workforce in primary care.

Answer:Thank you for your opinion. Tavabie J A's research corresponds to the literature [2], which is convenient for the author to search.

Research showed that the path of career planning and employment strategy based on deep learning in the information age can better help unemployed people understand career planning and formulate employment strategies to finally choose a correct job."

Answer:Thank you for your opinion. Based on this article, we have added relevant literature references to provide readers with an accurate basis for argumentation.

2 Career Planning and Employment Strategies in the Information Age of Deep Learning
This section is intended to summarize existing literature on deep learning in career planning, but it does not appear to serve this purpose.

Answer:Based on your suggestions, Chapter 2 of deep learning on career planning and employment strategies in the information age has been expanded and the latest literature has been introduced to achieve the purpose of research summary.
Additionally, a significant portion of this section is dedicated to explaining the Deep Learning Mode, which seems only tangentially related to the main topic.
Answer:According to your suggestion, there is indeed too much introduction to the deep learning mode part, and now some parts have been deleted to increase the focus on the topic of this article.

3 Experiment Design and Validation
The research methods described in this section lack theoretical grounding, and there is insufficient detail regarding the acquisition of data from the two schools, observation periods, and whether the experiments were conducted by the researchers or used publicly available data.

Answer:Thank you for your suggestion. The research method does lack a theoretical foundation. Now the experimental design has been supplemented with a detailed introduction to the data acquisition, observation period, experimental details, etc. of the two schools.
The manuscript does not provide details about the survey content or the number of responses collected.
If the two schools differ significantly in size or have not been adequately exposed to deep learning, the comparative results lack credibility.
Answer:Based on your comments, details regarding the survey content and number of responses collected are now provided, and the two schools are of similar size.
4Conclusions
The conclusions are overly simplistic and do not provide an adequate summary of the research or a comparison with existing studies.

Answer:Thank you for your suggestions. The conclusion has been rewritten, adding a full summary of the research and comparison with existing research to ensure the completeness of the conclusion.
