# Supplementary material for: Path of career planning and employment strategy based on deep learning in the information age
Source: PLoS One. 2024 Oct 15;19(10):e0308654. doi: 10.1371/journal.pone.0308654 (PMC11478877; doi:10.1371/journal.pone.0308654)
Supplement: Attachment — Submitted filename: Review.docx [file pone.0308654.s003.docx]

**Upload Reviewer Attachments for Manuscript Number PONE-D-23-39644
"Path of Career Planning and Employment Strategy Based on Deep Learning in the Information Age"**

First I appreciate your work and this is very recent issue which most of the nations are facing.

Try to include more citations in the introduction part, the way you explained the content in the introduction is really good but adding citation will enhance the quality of your work.

You mentioned that deep learning will help to enhance students abilities by estimating job market. Provide clarity on how deep learning helps students to find out jobs based on their abilities.

Need to provide detail explanation in the data analysis i.e which software tool you applied to analyse data.

Mention academic implications to provide clarity on how this study supports academicians and also provide theoretical implications.

Provide you suggestions to policy makers through this study.
